# Seroprevalence of Alkhurma and Other Hemorrhagic Fever Viruses, Saudi Arabia

**DOI:** 10.3201/eid1712.110658

**Published:** 2011-12

**Authors:** Ziad A. Memish, Ali Albarrak, Mohammad A. Almazroa, Ibrahim Al-Omar, Rafat Alhakeem, Abdullah Assiri, Shamsudeen Fagbo, Adam MacNeil, Pierre E. Rollin, Nageeb Abdullah, Gwen Stephens

**Affiliations:** Ministry of Health, Riyadh, Kingdom of Saudi Arabia (Z.A. Memish, M.A. Almazroa, I. Al-Omar, R. Alhakeem, A. Assiri, S. Fagbo, N. Abdullah, G. Stephens);; Riyadh Military Hospital, Riyadh (A. Albarrak);; Centers for Disease Control and Prevention, Atlanta, Georgia, USA (A. MacNeil, P.E. Rollin);; University of British Columbia, Vancouver, British Columbia, Canada (G. Stephens)

**Keywords:** viruses, arbovirus, Alkhurma hemorrhagic fever virus, dengue virus, Rift Valley fever virus, Crimean-Congo hemorrhagic fever virus, hemorrhagic fever, zoonotic infection, Saudi Arabia

## Abstract

A 2009 deployment of military units from several Saudi Arabian provinces to Jazan Province, Saudi Arabia, enabled us to evaluate exposure to Alkhurma, Crimean-Congo, dengue, and Rift Valley hemorrhagic fever viruses. Seroprevalence to all viruses was low; however, Alkhurma virus seroprevalence was higher (1.3%) and less geographically restricted than previously thought.

Jazan is a Red Sea port city on Saudi Arabia’s southern border with Yemen and the capital of Jazan Province. The region serves as an east–west portal from sub-Saharan Africa at Djibouti and a south–north route across the Yemeni frontier. It is a heavily traveled corridor for humans and animals entering Saudi Arabia, particularly during the annual Hajj pilgrimage. Malaria is endemic, and arbovirus infections are well described, most notably a 2000–2001 Rift Valley fever (RVF) outbreak on the Saudi Arabia–Yemen frontier. More than 880 confirmed RVF cases were reported. This outbreak was also notable for a case-fatality rate of 14% (*1*). Sporadic infections caused by Crimean-Congo hemorrhagic fever virus (CCHFV) occur as well. The virus is endemic across a wide geographic range, from the Middle East to Africa and central Asia (*2*). Dengue virus (DENV), a pathogen well known for large outbreaks and global circulation, also causes seasonal outbreaks in Saudi Arabia’s western provinces; most outbreaks occur farther north in the urban centers of Jeddah and Makkah (*3*).

In contrast, Alkhurma hemorrhagic fever is an emerging infectious disease that has been described mostly in Saudi Arabia. The responsible virus, first isolated in Jeddah by A.M. Zaki, has since been characterized as a distinct lineage of Kyasanur Forest disease virus, a tick-borne member of the family *Flaviviridae* (*4**–**6*). Epidemiologic studies of Alkhurma hemorrhagic fever virus (AHFV) have focused on Jeddah and Makkah, where outbreaks were first described; most were characterized by high rates of illness and death (*6*). Recent studies in Najran Province extended the spectrum of disease to include subclinical infection, which was far more frequent than severe disease (*7**,**8*). A recent report suggests wider geographic range for AHFV, with infections identified in 2 Italian tourists after they traveled to Egypt (*9*).

## The Study

In November 2009, Saudi military forces previously stationed in other parts of the country were deployed to Jazan Province. This situation enabled us to look at baseline arbovirus seroprevalence in a group of new arrivals, stratified by province of origin. During May 8–12, 2010, we enrolled 1,026 soldiers in 5 Jazan administrative units near the border with Yemen in a study to evaluate serologic reactivity to AHFV, CCHFV, DENV, and RVFV. After receiving consent and assigning numeric identifiers to anonymize data, we used questionnaires to record Jazan arrival dates, home province, previous administrative residence, health histories, vector exposures, and other risk factors. Answers were reviewed onsite, and a 5-mL blood sample was collected. Serum samples from each soldier were labeled, archived, frozen, stored at –80°C, and transferred to the Ministry of Health central laboratory in Riyadh for testing. Questionnaire and testing data were entered in Epi Info software (wwwn.cdc.gov/epiinfo) and then transferred to SPSS version 19.0 (IBM, Somers, NY, USA) for analysis.

A total of 197 (19%) enrolled soldiers reported symptomatic illness during deployment, 49 (25%) of whom were hospitalized. Reported signs and symptoms were fever (n = 81), rash (n = 50), and musculoskeletal complaints (n = 128). A diagnosis of malaria was recorded for 27 febrile soldiers and dengue fever for 1. Illnesses of the remaining soldiers were undiagnosed. As expected given the number of malaria cases, reported arthropod exposures favored mosquitoes over ticks, with 875 (85%) soldiers reporting mosquito contact compared with 153 (15%) reporting tick encounters. Thirty-seven (3%) soldiers reported contact with livestock carcasses, blood, or body fluids.

Serologic testing was completed for 1,024 soldiers; initial screening by IgG to each of the 4 viruses was followed by IgM testing of all IgG-reactive samples. Dengue antibodies were tested by using PanBio ELISA IgG (E-DEN02G; Inverness Medical Innovations, Sinnamon Park, QLD, Australia) and IgM (E-DEN01M; Inverness Medical Innovations) following manufacturer recommendations and protocols. IgG and IgM testing for AHFV, CCHV, and RVFV was done with Centers for Disease Control and Prevention (Atlanta, GA, USA) reagents and protocols by using cell culture–derived antigens (*7**,**10**,**11*). Briefly, the ELISA antigens used to coat plates (for IgG) or detect captured IgM were produced by infecting Vero E6 cells with respective reference virus strains or by using uninfected cells for control. Each sample was tested at 4 dilutions (100, 400, 1,600, and 6,400). IgG reactivity/IgM nonreactivity was considered evidence of past infection; concurrent IgG/IgM reactivity was interpreted as infection within the previous 6 months. IgG-seropositive persons without histories of illness were considered to have had subclinical or very mild infection.

Forty reactive serum samples were identified, for a combined seroprevalence of 3.9 cases/100 soldiers tested: RVF (n = 20), AHFV (n = 13), CCHV (n = 6), and DENV (n = 1) (Table). One soldier who had a positive test result for IgG and IgM to RVFV had a rash but no history of fever. No soldiers with AHFV, CCHFV, or DENV IgM were identified. We did not observe cross-reactivity of antibodies often seen with flaviviruses, and no person had positive test results against >1 antigen.

**Table:** Serologic status of 1,024 soldiers evaluated for IgG and IgM ELISA reactivity against AHFV, CCHF, DENV, and RVFV antigens, Saudi Arabia, 2009*

Virus	No. (%) IgG reactive	No. IgM reactive	No. symptomatic	Seroprevalence/100 soldiers tested
RVFV	20 (2.0)	1	1 (rash)	1.95
AHFV	13 (1.3)	0	0	1.27
CCHF	6 (0.6)	0	0	0.58
DENV	1 (0.1)	0	0	0.1
Total	40 (3.9)	1	1	3.9

*AHFV, Alkhurma hemorrhagic fever virus; CCHF, Crimean-Congo hemorrhagic fever; DENV, dengue virus; RVFV, Rift Valley fever virus.

Analysis of the province of origin provided AHFV epidemiologic information (Figure). Two hundred sixty-eight (26%) soldiers were transferred to Jazan from Tabouk. This northern district accounted for most seropositive soldiers (8/13), 6 of whom might have resided in or visited a previously known AHFV-endemic region before transfer. Three AHFV-seropositive soldiers arrived from the Eastern Region; none had a previous residence or travel history elsewhere. In contrast, most RVFV-seropositive soldiers (18/20) and all 6 CCHV-seropositive soldiers had previously worked and resided in Jazan or other previously affected region. The only RVFV IgM-seropositive soldier listed a home residence and previous workplace in an AHFV-endemic region.

**Figure:**
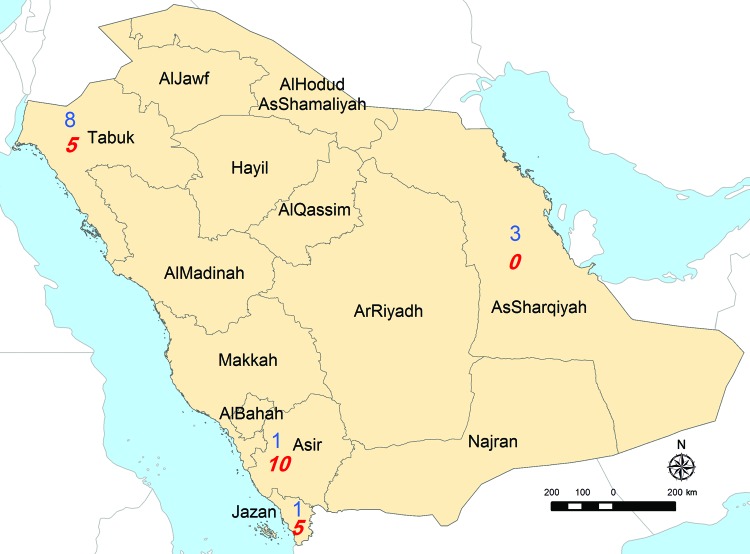
Numbers of soldiers with seropositive test results distributed according to Saudi Arabian province before transfer to Jazan, 2009. Blue (n = 13), seropositive for Alkhurma hemorrhagic fever virus; red (n = 20), seropositive for Rift Valley fever virus. Map courtesy of Al Zahrani.

## Conclusions

Although the full geographic distribution and severity of AHFV infection is still being characterized, data from this study imply a wider range of endemicity than previously reported. For instance, most seropositive persons came from Tabouk and Eastern Directorates. This finding is not surprising knowing the large geographic distribution of the suspected tick vectors, *Ornithodoros savignyi* and *Hyalomma dromedarii* (*12**,**13*); however, neither hemorrhagic fever nor AHFV infection has been reported from either region. Nor did any AHFV-seropositive soldier disclose a history of severe illness, consistent with a 2006–2009 Narjan outbreak study that found seropositive status to be highly correlated with mild or asymptomatic infection. Although additional studies are required to further characterize Alkhurma’s natural history, case-fatality rates of 25%–30% reported after earlier outbreaks in Jeddah and Makkah appear to be overestimates (*14*).

In contrast, all CCHFV-seropositive and RVFV-seropositive soldiers had resided in Jazan or an adjacent region previously endemic for those viruses. Apart from the reported episodes of CCHF surrounding the importation of infected livestock or ticks, the epidemiology and distribution of this virus in Saudi Arabia are unclear. Our results showed that it is circulating at least in the western part of the country. A 2% RVFV seroprevalence in our study, although not necessarily generalizable to the entire population, is not surprising given the large number of cases during the 2000–2001 outbreak (*1**,**15*). Low dengue seroprevalence contrasts with large recurring outbreaks in Jeddah and Makkah and may have implications for future prevention strategies.

Although IgM testing was not done on IgG-seronegative samples, the absence of compatible illness in surveyed soldiers suggests IgG was adequate for surveillance screening. Other potential limitations include unknown specificity of some tests used to survey these viruses and recall accuracy of travel histories or residences reported by the study’s participants. Nonetheless, this study provides systematic evidence that Alkhurma, Crimean-Congo hemorrhagic fever, dengue, and Rift Valley fever viruses are endemic to western, if not all provinces of, Saudi Arabia. A better understanding of their ecology, natural history, and epidemiology is needed to assess the potential risks to public health.
